# Recent advances in CRISPR/Cas9 and applications for wheat functional genomics and breeding

**DOI:** 10.1007/s42994-021-00042-5

**Published:** 2021-04-15

**Authors:** Jun Li, Yan Li, Ligeng Ma

**Affiliations:** 1 College of Life Sciences, State Key Laboratory of North China Crop Improvement and Regulation, Hebei Agricultural University, Baoding, 071001 Hebei China; 2 College of Life Sciences, Beijing Key Laboratory of Plant Gene Resources and Biotechnology for Carbon Reduction and Environmental Improvement, Capital Normal University, Beijing, 100048 China

**Keywords:** Wheat, CRISPR/Cas9, Genome editing, Functional genomics, Breeding

## Abstract

Common wheat (*Triticum aestivum* L.) is one of the three major food crops in the world; thus, wheat breeding programs are important for world food security. Characterizing the genes that control important agronomic traits and finding new ways to alter them are necessary to improve wheat breeding. Functional genomics and breeding in polyploid wheat has been greatly accelerated by the advent of several powerful tools, especially CRISPR/Cas9 genome editing technology, which allows multiplex genome engineering. Here, we describe the development of CRISPR/Cas9, which has revolutionized the field of genome editing. In addition, we emphasize technological breakthroughs (e.g., base editing and prime editing) based on CRISPR/Cas9. We also summarize recent applications and advances in the functional annotation and breeding of wheat, and we introduce the production of CRISPR-edited DNA-free wheat. Combined with other achievements, CRISPR and CRISPR-based genome editing will speed progress in wheat biology and promote sustainable agriculture.

## Introduction

Common wheat is a keystone crop species. It is grown in many different environments, providing most humans with around 20% of their calories and protein (Uauy et al. 2017); thus, it occupies an important position in food security. As the global population increases, improving the yield of wheat is critical to ensure future availability. Geneticists have exploited natural or artificial wheat variations for breeding. Indeed, conventional breeding approaches have played a major role in increasing grain yields and quality based on broad genetic variations in wheat (Nadolska-Orczyk et al. 2017). However, wheat is an allohexaploid (2*n* = 6 × = 42, AABBDD); it harbors three closely related subgenomes inherited from three homoeologous ancestors (Petersen et al. 2006). Thus, most wheat genes have three similar but not identical copies, with functional redundancy and complementarity among the A, B, and D genomes. As a result, the probability of the simultaneous mutation of genes in the A, B, and D genomes by natural processes or induced mutagenesis is very low. Therefore, the complex polyploid nature of wheat has hindered the development of functional genomics and breeding, especially compared to other cereals, such as rice and maize.

Several genome editing technologies with the ability to change the code of life with high specificity have been developed recently (Li et al. 2019). Advances in genome editing have revolutionized life science, including plant science. The clustered regularly interspaced short palindromic repeats (CRISPR)/CRISPR-associated protein 9 (Cas9) system offers several advantages, including simplicity, versatility, high efficiency, and the ability to work with multiple targets simultaneously (multiplexing); it has surpassed other genome editing tools, becoming the most widely used gene editing technology in the world (Doudna and Charpentier 2014). Thus far, CRISPR/Cas9 has been used to create various targeted mutations in a broad range of living organisms (Char and Yang 2020; Gürel et al. 2020).

Application of the CRISPR/Cas9 system requires the DNA sequences of the target genes. Given the availability of the annotated wheat genome and the elucidation of a growing number of genes controlling important agronomic traits in other plants, it is easy to isolate orthologous genes in wheat based on homology-based cloning. In addition, CRISPR/Cas9 allows researchers to target multiple homoeoalleles simultaneously and it enables the production of targeted mutations in all copies of a gene; thus, the system holds great promise in the characterization of genes endowing important agronomic traits in polyploid wheat. Furthermore, it has been used to modify multiple genes controlling different agronomic traits in wheat. This technology will bring a new dawn to wheat biology and breeding programs. In this review, we briefly outline the utilization of the CRISPR/Cas9 system, with an emphasis on the most important breakthroughs thus far. We also summarize recent applications of genome editing in wheat. Finally, we discuss the future prospects of CRISPR/Cas9 genome editing for wheat improvement.

## Genome editing with CRISPR/Cas9

### Mechanism of genome editing

Genome editing technology generates site-specific double-strand breaks (DSBs) in the targeted genomic sequence using programmable sequence-specific nucleases (SSNs), and then exploits endogenous DSB repair mechanisms to generate a variety of mutations in the target region. Three types of SSNs are used to introduce DSBs at selected sites: zinc-finger nucleases, transcription activator-like effector nucleases, and CRISPR/Cas9 (Kim and Kim 2014; Zhan et al. 2020). Subsequently, the DSBs are mainly repaired via two pathways, nonhomologous end joining (NHEJ) and homologous recombination (HR) (Symington and Gautier 2011). In NHEJ, two broken ends are simply re-ligated, producing insertions and/or deletions (indels) in the target site. When homologous donor sequences are present at the DSBs, HR may be used, and desired gene modifications occur (Chen et al. 2019; Anzalone et al. 2020). Overall, SSN-induced DSBs are repaired more frequently by the NHEJ pathway than by the HR pathway (Carroll 2014; Gao 2021).

### CRISPR/Cas9

CRISPR/Cas systems provide a defense against foreign plasmids or viral DNA elements in bacteria and archaea. They are divided into six types based on the assortment of *cas* genes and nature of the interference complex (Hille et al. 2018). Three components, mature crRNA, tracrRNA, and Cas9, are responsible for cleaving the invading elements in type II CRISPR/Cas systems. To simplify the system, a dual tracrRNA:crRNA was designed as a single guide RNA (sgRNA) to direct the production of DSBs by Cas9 in vitro (Jinek et al. 2012). Subsequently, an RNA-programmable genome editing tool, CRISPR/Cas9, was developed to create targeted mutations (Cong et al. 2013; Mali et al. 2013; Zhang et al. 2019c).

**Fig. 1:**
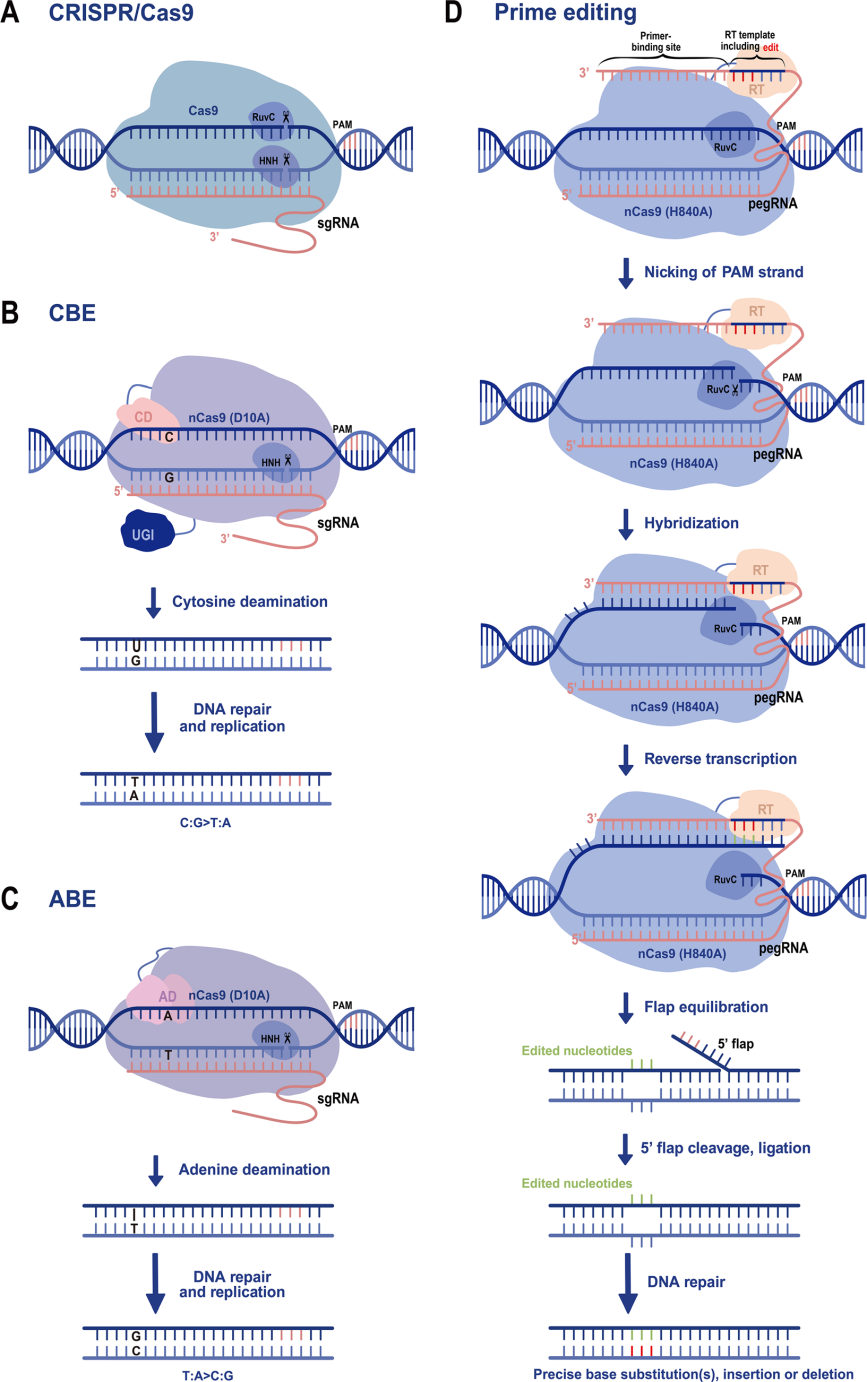
Overview of CRISPR/Cas9 and CRISPR/Cas9-based precise genome editing. **A** CRISPR/Cas9-mediated genome editing. CRISPR/Cas9 contains two components: Cas9 and a sgRNA. Guided by the 20-nucleotide sequence within sgRNAs, Cas9 cleaves the DNA double-strand, generating a blunt-end DSB. **B** Mechanism of CBE-mediated C-to-T base editing. The CBE is comprised of nCas9 (D10A) fused with two proteins [cytosine deaminase (CD) and uracil DNA glycosylase inhibitor (UGI)] and a sgRNA. Guided by the 20-nucleotide sequence within sgRNAs, CD converts C to U within the window. With DNA repair and replication, it generates C-to-T substitutions. **C** Mechanism of ABE-mediated A-to-G base editing. The ABE is comprised of nCas9 (D10A) fused with adenine deaminase (AD, TadA-TadA* heterodimers), and a sgRNA. Guided by the 20-nucleotide sequence within sgRNAs, AD converts A to I within the window. With DNA repair and replication, it generates A-to-G substitutions. **D** Mechanism of prime editing. It contains nCas9 (H840A) fused with reverse transcriptase (RT) and pegRNA. Guided by the 20-nucleotide sequence within pegRNAs, RT primes new DNA containing the desired editing at the targeted site. After flap equilibration, cleavage, ligation, and DNA repair, the desired editing is incorporated

CRISPR/Cas9 contains two major components: a sgRNA, which is responsible for recognizing target DNA, and the Cas9 endonuclease, which is responsible for generating DSB at predesigned target DNA site (Fig. 1A). Cas9 from *Streptococcus pyogenes* (*SpCas9*) was the first well-characterized RNA-guided endonuclease. It is a multifunctional protein that contains two nuclease domains: the HNH domain and RuvC-like domain. Each of them cuts one DNA strand, generating blunt-end DSBs; this triggers endogenous DNA repair systems, resulting in targeted mutants. The only prerequisite for applying CRISPR/Cas9 to a given site is the presence of a protospacer-adjacent motif (PAM; NGG for *SpCas9*) next to the sequence of interest. For different target sites, Cas9 is constant; we can only change the guide sequence in the sgRNA.

### Base editing

Base editing, borrowed from CRISPR, is a precise genome editing approach. It generates targeted point mutations without DSBs, foreign donor templates, or HR (Komor et al. 2016; Gaudelli et al. 2017). Current base editors usually contain a sgRNA, and a catalytically impaired Cas9 nuclease [dead Cas9 (dCas9) or Cas9 nickase (Cas9n)] fused with ssDNA deaminase. The sgRNA guides the modified Cas9-deaminase to the target locus, generating ssDNA R-loop that is exposed and accessible to the deaminase (Anzalone et al. 2020). Based on the different kinds of deaminase, there are two major groups of DNA base editors: cytidine base editors (CBEs) and adenine base editors (ABEs).

With CBEs, cytidine deaminase is used to convert cytidine (C) to uridine (U) within the editing window, creating a mismatched base pair with guanine (G) on the opposite strand (Fig. 1B). However, the U intermediate is mutagenic; most organisms have evolved a uracil base excision repair (BER) pathway to excise U from genomic DNA with uracil DNA N-glycosylase. Therefore, uracil glycosylase inhibitor protein (UGI) is used to impede uracil excision, increasing the C-to-T editing efficiency of CBEs (Komor et al. 2016; Nishida et al. 2016). This approach was first used in yeast and human cells and then applied to a variety of plants, including wheat, rice, maize, tomato, and *Arabidopsis* (Zong et al. 2017; Shimatani et al. 2017; Chen et al. 2017).

Theoretically, dCas9/nCas9 fused with an adenosine deaminase would yield an ABE. However, there is no known natural deaminase that deaminates adenine in DNA. By extensive directed evolution and protein engineering of *Escherichia coli* tRNA adenine deaminase (TadA), researchers produced a deaminase variant (TadA*) that can deaminate adenine (A) in DNA. TadA-TadA* heterodimers were fused with dCas9/nCas9 to generate an ABE (Fig. 1C); adenine is deaminated to inosine (I), treated as G by the polymerase, converting AT to GC base pairs in human cells (Gaudelli et al. 2017). Several groups have shown that ABEs can be applied to plants, including wheat, rice, potato, *Arabidopsis*, and *Brassica napus* (Kang et al. 2018; Zong et al. 2018).

Many important agronomic traits involve single-nucleotide variants (Zhao et al. 2011; Hu et al. 2015). Therefore, precise editing of a single nucleotide in plants is a desirable and powerful means of accelerating crop improvement. Base editors (CBEs and ABEs) can efficiently mediate all four transition mutations (C → T, A → G, T → C, and G → A) at targeted loci; this will undoubtedly facilitate basic research and breeding in plants.

### Prime editing

Prime editing, borrowed from CRISPR, is another precise genome editing method that can generate all 12 types of base substitutions, targeted small insertions, deletions, and combinations of these editing results in the target site. The prime editor mainly consists of a catalytically impaired Cas9 (Cas9n, H840A) fused with an engineered reverse transcriptase and a prime editing guide RNA (pegRNA) (Fig. 1D). The latter contains a primer-binding site at the 3′ end of the sgRNA and a reverse transcriptase template, specifying the target site and also encoding the desired sequence edit (Anzalone et al. 2019).

In the prime editing system, the complex binds to the target DNA site and nicks the PAM-containing strand, generating a 3′ end. Then, it hybridizes to the primer-binding site of the pegRNA and primes reverse transcription of the template containing the desired edits. After equilibration between the edited 3′ flap and the unedited 5′ flap, 5′ flap excision, ligation, and DNA repair, DNAs are stably edited in the desired manner (Anzalone et al. 2019). It was first used to correct mutations in human cells and has since been successfully applied to rice plants and wheat protoplasts (Li et al. 2020a; Lin et al. 2020; Xu et al. 2020; Hua et al. 2020). This search-and-replace method is a versatile and precise genome editing tool that does not require DSBs, or donor DNA templates.

## Recent applications of genome editing in wheat

When just one homolog mutates, no mutant phenotype might be observed due to masking by other homologs (Borrill et al. 2015). This genetic redundancy and complementarity has hindered the development of wheat biology. Now that CRISPR/Cas9 has been used to target multiple homoeoalleles simultaneously (Table 1), it will accelerate progress in functional genomics and molecular breeding in wheat.

**Table 1:** List of wheat endogenous gene(s) targeted using CRISPR in the literature

Targeted gene(s)	Genome editing method	PAM type(s)	Repair result	Delivery	Efficiency	Reference
*MLO*	CRISPR/Cas9	CCN	Indels	Particle bombardment	5.6%	Wang et al. (2014)
*GASR7*, *GW2*, *DEP1*, *NAC2*, *PIN1*, *LOX2*	CRISPR/Cas9	CCN, NGG	Indels	Particle bombardment	1.0–9.5%	Zhang et al. (2016)
*EDR1*	CRISPR/Cas9	CCN	Indels	Particle bombardment	N.A	Zhang et al. (2017)
*gliadin*	CRISPR/Cas9	CCN	Indels	Particle bombardment	62.3–75.1%	Sanchez-Leon et al. (2018)
*PDS*	CRISPR/Cas9	CCN, NGG	Indels	*Agrobacterium*	11–17%	Howells et al. (2018)
*GW2*, *Lpx-1*, *MLO*	CRISPR/Cas9	CCN, NGG	Indels	Particle bombardment	3–32%	Wang et al. (2018a)
*CKX2-1*, *GLW7*, *GW2*, *GW8*	CRISPR/Cas9	CCN, NGG	Indels	*Agrobacterium*	10%	Zhang et al. (2019d)
*Qsd1*	CRISPR/Cas9	NGG	Indels	*Agrobacterium*	37.5%	Abe et al. (2019)
*DA1*, *DA2*, *NCED1*, *LPR2*	CRISPR/Cas9	CCN, NGG	Indels	*Agrobacterium*	20.8–54.2%	Zhang et al. (2019b)
*GW7*	CRISPR/Cas9	NGG	Indels	Particle bombardment	1.1–8.3%	Wang et al. (2019)
*Ms45*	CRISPR/Cas9	NGG	Indels	*Agrobacterium*	N.A	Singh et al. (2018)
*Ms1*	CRISPR/Cas9	CCN, NGG	Indels	*Agrobacterium*	5%	Okada et al. (2019)
*NP1*	CRISPR/Cas9	NGG	Indels	Particle bombardment	14.0–54.2%	Li et al. (2020b)
*Ms2*	CRISPR/Cas9	CCN, NGG	Indels	*Agrobacterium*	2.3–8.27%	Tang et al. (2020)
*MTL*, *Waxy*	CRISPR/Cas9	CCN, NGG	Indels	*Agrobacterium*	8.9–71.3%	Liu et al. (2020a)
*Q*	CRISPR/Cas9	NGG	Indels	*Agrobacterium*	44.0–45.6%	Liu et al. (2020b)
*CENH3*	CRISPR/Cas9	CCN, NGG	Indels, restored frameshift	Particle bombardment	N.A	Lv et al. (2020)
*SBEIIa*	CRISPR/Cas9	CCN, NGG	Indels	Particle bombardment	38.5–66.7%	Li et al. (2020c)
*LOX2*	BE3	NGG	C-to-T conversion	Particle bombardment	1.25%	Zong et al. (2017)
*ALS*, *ACCase*	BE3	CCN, NGG	C-to-T conversion	Particle bombardment	2.5%	Zhang et al. (2019a)
*ALS*, *MTL*	A3A-PBE	NGG	C-to-T conversion	Particle bombardment	16.7–22.5%	Zong et al. (2018)
*DEP1*, *GW2*	ABE7.10	NGG	A-to-G conversion	Particle bombardment	0.4–1.1%	Li et al. (2018)
*GW2*, *LOX2*, *MLO*, *GASR7*, *DME*	Prime editing	NGG	Point mutations, targeted insertions and deletions	Particle bombardment	N.A	Lin et al. (2020)

*NA* not available

### Improving wheat yields and grain quality

As the world’s population grows, wheat yields will need to substantially increase to ensure global food security. This fact has pushed scientists to investigate and breed innovative wheat varieties. Using CRISPR/Cas9, many negative regulatory genes have been knocked out to improve wheat yields and quality. For example, *GASR7* is a gibberellin-regulated gene that controls grain length in rice. Simultaneous targeting of all three *TaGASR7* homoeologs significantly elevated the thousand kernel weight, irrespective of the varietal background (Zhang et al. 2016). Similarly, *GW2*, encoding a RING-type E3 ligase that controls rice grain weight, was knocked out to increase the length and width of wheat grains and, hence, grain yields (Wang et al. 2018b; Zhang et al. 2018).

To meet different customers’ needs, grain quality is an important trait that should be improved. For example, gluten proteins, encoded by *gliadin* genes in wheat, are major factors triggering celiac disease in genetically predisposed individuals. Researchers designed two sgRNAs to target the conserved region of *a-gliadin* genes; they generated low-gluten wheat, for which the immunoreactivity was reduced by 85% (Sanchez-Leon et al. 2018). Moreover, targeted mutagenesis of *TaSBEIIa* by CRISPR/Cas9 successfully generated high-amylose wheat with a significantly increased resistant starch content (Li et al. 2020d). Therefore, wheat yields and quality traits can be successfully improved using CRISPR/Cas9.

### Speeding wheat hybrid seed production

Heterosis is widely exploited to improve crop productivity and other agronomic traits. However, due to wheat’s strong inbreeding habit, it has been a great challenge to develop male-sterile wheat lines for the production of hybrid seed (Singh et al. 2018). The identification and manipulation of male sterility genes is the first step to generate novel male-sterile wheat mutants. Given the recent molecular identification of male fertility genes in plants, it is possible to clone the homoeoalleles using homology-based cloning, and then create male-sterile hexaploid wheat lines using CRISPR/Cas9. Indeed, great progress has been achieved recently. For instance, *NP1* encodes a putative glucose-methanol-choline oxidoreductase that is required for male sterility in rice (Chang et al. 2016). Using an optimized CRISPR/Cas9 system, our group simultaneously disrupted three *TaNP1* homoeoalleles in wheat. The resulting *Tanp1* triple mutants showed complete male sterility (i.e., produced no pollen) (Li et al. 2020b). Similarly, the targeted knockout of *Ms1*, which is responsible for pollen exine development and male fertility (Tucker et al. 2017; Wang et al. 2017b), produced complete male sterility in commercial wheat cultivars (Okada et al. 2019). These male sterility mutants could accelerate hybrid breeding of wheat. Very recently, *TaCENH3α* was edited using CRISPR/Cas9; paternal haploid inducer wheat lines were generated with an induction rate of ~ 7% (Lv et al. 2020). This could be used for additional new breeding technologies and paves the way for reducing the cost of goods in wheat seed production.

### Increasing disease resistance in wheat

Diseases induced by fungi, bacteria, and viruses could reduce wheat yields and quality dramatically. CRISPR/Cas9 has been used to knock out disease-susceptibility genes to generate disease-resistant wheat. In plants, a loss of function of MILDEW-RESISTANCE LOCUS (*MLO*) confers broad-spectrum resistance to powdery mildew (Gil-Humanes and Voytas 2014). This makes *MLO* an ideal target for CRISPR/Cas9 to enhance resistance to powdery mildew. Researchers knocked out all six *MLO* alleles in wheat; they produced a *Tamlo* triple mutant showing increased resistance to powdery mildew disease (Wang et al. 2014). Similarly, the gene encoding enhanced disease resistance1 (EDR1), a negative factor against powdery mildew defenses, has been simultaneously modified using CRISPR/Cas9, generating wheat with improved powdery mildew resistance (Zhang et al. 2017). Thus, CRISPR/Cas9 is an important means to enhance disease resistance in wheat.

### Generating CRISPR-edited DNA-free wheat

CRISPR/Cas9 is widely used to improve agricultural traits by knocking out unwanted genes or genes conferring undesirable phenotypes. However, this process usually involves transgenic intermediates, which causes regulatory concerns and is not accepted worldwide (Zhang et al. 2020). For public acceptance, gene removal or bypassing foreign elements to edit endogenous genes is a good choice (He and Zhao 2020). Based on the reagents needed for CRISPR-mediated editing, there are two main ways to produce CRISPR-edited DNA-free plants.

In the vector-based method, a vector is delivered into wheat callus using *Agrobacterium* or particle bombardment. It then integrates into the genome and the encoded genome editing elements are expressed, enabling targeted gene knockout. Targeted knockout wheat with foreign DNA is generated in the T0 generation. Ultimately, the foreign DNA can be segregated by selfing and crossing. For example, researchers created a triple-knockout mutant of *TaQsd1* via *Agrobacterium*-delivered CRISPR/Cas9. The mutant was then crossed with wild-type wheat plants, producing transgene-free triple-recessive *TaQsd1* mutants that exhibited longer seed dormancy (Abe et al. 2019). Similarly, a marker-free wheat mutant was obtained among the offspring of T0 plants (Wang et al. 2017a).

Sometimes, vectors are not integrated into the genome; instead, they may transiently express their encoded genome editing elements to knock out genes. A targeted gene-modified plantlet without foreign DNA is generated in the T0 generation. This approach has been reported in wheat for the first time. Researchers delivered vectors containing CRISPR/Cas9 elements into wheat callus through particle bombardment; the plantlet was subsequently regenerated without antibiotic selection. This transient expression-based CRISPR/Cas9 system produced transgene-free, homozygous mutants (Zhang et al. 2016). In addition, transgene-free wheat carrying nucleotide substitutions have been generated by transiently expressing CBEs or ABEs (Zong et al. 2017; Li et al. 2018).

In the non-vector method, Cas9 and sgRNAs are transcribed in vitro and then delivered into immature wheat embryos through particle bombardment. DNA-free genome-edited wheat plants have been generated. Though the editing efficiency was lower, the specificity was higher than with a vector-based system (Zhang et al. 2016). Moreover, nCas9-PBE mRNA and sgRNA were transcribed in vitro and delivered into immature wheat embryos. DNA-free base editing at TaALS-P174 was obtained, endowing wheat with resistance to the herbicide nicosulfuron (Zhang et al. 2019a). In addition, Cas9 can be expressed in vitro and assembled with the sgRNA into a Cas9/sgRNA ribonucleoprotein, which is delivered into immature wheat embryos by particle bombardment. The ribonucleoprotein cleaves the target site immediately and is quickly degraded, generating DNA-free edited wheat (Liang et al. 2017).

The final CRISPR-edited DNA-free products are similar to natural and artificial mutants, which are not subject to GMO regulations. We believe that this is the direction of future breeding, and it will play a vital role in realizing sustainable agriculture in the future.

## Concluding remarks and future perspectives

Though large genome and complex polyploid nature have hindered the development of wheat genetic engineering and breeding in the past, several powerful tools are now available to advance wheat biology (Li et al. 2020c). In particular, the development of CRISPR/Cas9 technology has been widely used in wheat genome editing. This technology allows multiplex genome engineering, which has enabled the production of loss-of-function triple wheat mutants; thus, it is a powerful tool for introducing desired traits conferred by a loss-of-function mutation into commercial cultivars via NHEJ. For example, a recessive genic male-sterile (GMS) mutant has several advantages for hybrid wheat production (Li et al. 2020b). As additional genes required for genic male sterility are identified, CRISPR/Cas9-mediated disruption of these genes will enable the rapid production of male-sterile wheat. This represents a promising method for manipulating recessive sterility genes to capture heterosis in wheat.

Some valuable alleles are often caused by one or several SNPs or defined insertion/deletions. The introduction of such valuable alleles into commercial cultivars requires 8–10 years by crossing and back-crossing to eliminate unexpected linked traits (Chen et al. 2019). Moreover, breeding is a complicated matter. Sometimes it is not enough to create a good variety by simply modifying one or two genes. For example, five *Puccinia graminis* f. sp. *Tritici* (*Pgt*) resistance genes have been introduced into bread wheat, conferring wheat with broad-spectrum resistance in the field (Luo et al. 2021). Nowadays, CRISPR-mediated precise genome editing is a useful means to achieve these targeted substitutions and replacements by modifying endogenous genes without introducing linkage drag; it can also introduce new alleles (segregating as a single locus) into a predetermined genomic site. Thus, this approach could accelerate the breeding process.

Though precise gene modification has been achieved in *Arabidopsis*, rice, maize, and tomato, it is only feasible in a few laboratories with low efficiency (Li and Xia 2020). To date, except for base editing, precise editing in wheat has not been achieved (Gil-Humanes et al. 2017; Lin et al. 2020). Therefore, precise gene modification in wheat remains a challenge. Given the increased focus of researchers on the mechanism of HR, we believe that precise gene editing via HR will be used for wheat breeding in the near future. The alternative is the dominant repair pathway—NHEJ, which has been exploited to generate gene replacements and gene knock-ins in rice (Li et al. 2016; Dong et al. 2020). It is a promising method to achieve precise genome modification in wheat, and it may facilitate wheat breeding by modifying gene functions or introducing new alleles into a predetermined genomic safe harbor.

Transgenerational CRISPR/Cas9 activity has been used to modify multiple target sites in tomato and wheat (Rodriguez-Leal et al. 2017; Wang et al. 2018a). This suggests that valuable, desired phenotypes in elite wheat germplasms, which are recalcitrant to transformation, could be induced by crossing with lines carrying CRISPR/Cas9 elements. In addition, wheat genes have been successfully edited via pollination using CRISPR/Cas9-transgenic maize as a haploid inducer (Kelliher et al. 2020; Budhagatapalli et al. 2020). Such haploid induction-mediated genome editing would not only reduce the genotype dependence on site-specific mutagenesis in wheat, but also provide a path to produce transgene-free gene-edited inbred wheat lines. Collectively, these technologies will accelerate wheat breeding.

Some studies have reported that although CRISPR/Cas9 can cleave a target site, sometimes it also cleaves sites with a few mismatches to the target site. This off-target effect is a major concern in gene therapy, but this issue might not be a barrier in plant biotechnology. The putative off-target mutation could be eliminated through back-crossing or crossing with wild-type plants. Moreover, it is advisable to design target sites using web-based tools to reduce off-target mutations by leveraging computation.

The fields of genome editing and wheat biology are attracting more and more excellent scientists, and as the number of available CRISPR/Cas platforms increases, additional tools for precisely fine-tuning gene expression will become available in wheat. Combined with other achievements, including the production of high-quality genome sequences and improved transgenic methods, CRISPR and CRISPR-based genome editing will bring functional genomics and rational design-based molecular breeding of polyploid wheat to the forefront of wheat biology. We believe that transgene-free, gene-edited wheat will play a critical role in addressing environmental issues while promoting sustainable agriculture. Significantly, it is not a replacement for traditional breeding; it is just one of the methods advancing wheat breeding programs and accelerating wheat biology.
